# A Molecular Tetrahedral Cobalt–Seleno-Based Complex as an Efficient Electrocatalyst for Water Splitting

**DOI:** 10.3390/molecules26040945

**Published:** 2021-02-10

**Authors:** Ibrahim Munkaila Abdullahi, Jahangir Masud, Polydoros-Chrisovalantis Ioannou, Eleftherios Ferentinos, Panayotis Kyritsis, Manashi Nath

**Affiliations:** 1 Department of Chemistry, Missouri University of Science & Technology, Rolla, MO 65409, USA; ima284@mst.edu; 2 Electrochemical Process Development, Energy & Environmental Research Centre, Grand Forks, ND 58202, USA; jmasud@undeerc.org; 3 Department of Chemistry, National and Kapodistrian University of Athens Panepistimiopolis, 15771 Athens, Greece; polydwros25@gmail.com (P.-C.I.); l_ferentinos@yahoo.gr (E.F.)

**Keywords:** cobalt complex, electrocatalysis, water splitting, hydrogen evolution, oxygen evolution

## Abstract

The cobalt–seleno-based coordination complex, [Co{(SeP^i^Pr_2_)_2_N}_2_], is reported with respect to its catalytic activity in oxygen evolution and hydrogen evolution reactions (OER and HER, respectively) in alkaline solutions. An overpotential of 320 and 630 mV was required to achieve 10 mA cm^−2^ for OER and HER, respectively. The overpotential for OER of this **CoSe_4_**-containing complex is one of the lowest that has been observed until now for molecular cobalt(II) systems, under the reported conditions. In addition, this cobalt–seleno-based complex exhibits a high mass activity (14.15 A g^−1^) and a much higher turn-over frequency (TOF) value (0.032 s^−1^) at an overpotential of 300 mV. These observations confirm analogous ones already reported in the literature pertaining to the potential of molecular cobalt–seleno systems as efficient OER electrocatalysts.

## 1. Introduction

Over the past few decades, research interest in materials design and synthesis, the applications of which can be channeled towards sustainable energy generation and storage, has increased tremendously, due to the continuing depletion of fossil fuels. Research work on these materials has primarily focused on identifying earth-abundant non-precious metal-based resources affording sustainable energy conversion from renewable sources such as the sun, wind and water. Among these, water splitting capable of generating clean hydrogen fuel on-demand has attracted considerable interest due to its wide range of applicability in various technologies including fuel cells, solar-to-fuel energy conversion, and water electrolyzers [1,2]. Electrocatalytic water splitting comprises two primary reactions occurring simultaneously: a hydrogen evolution reaction (HER) at the cathode, and an oxygen evolution reaction (OER) at the anode. The latter reaction is an energy intensive process involving multi-step proton-coupled electron transfer steps, and is thermodynamically less favorable, making it a major barrier for the advancement of these technologies [3].

Owing to the slow kinetics of oxygen evolution reactions (OER), catalysts are typically used to reduce the activation energy barrier as well as stabilize intermediate adsorption on the catalyst surface. Among these, precious metal-based systems such as iridium and ruthenium oxides are considered as state-of-the-art OER catalysts, while platinum-based materials are best for HER, with only a moderate activity towards OER. The use of precious metal based-oxides, however, is a severely limiting factor for widespread commercial applications due to their high cost and scarcity [1]. Consequently, the search for robust and efficient OER and HER catalysts based on abundant non-precious transition elements has attracted considerable attention. Numerous types of such catalysts have been investigated with an aim to replace precious metal-based electrocatalysts without compromising either catalytic efficiency or stability.

Among the transition metals, cobalt-based electrocatalysts have emerged as an attractive class of non-precious metal-based catalysts for electrochemical water oxidation reactions, due to their abundance, stability, and more importantly superior catalytic performance [4]. Some of the most promising cobalt-based compounds identified as cost-effective and efficient catalysts for OER and HER are oxides [5,6,7] and chalcogenides (Co_x_E_y_; E = S, Se, Te) [8,9,10,11,12]. A lot of cobalt selenides of the generic formula Co_x_Se_y_ have been reported to exhibit promising electrocatalytic activity for either HER or OER or both. Huge progress has also been made in efforts to develop bifunctional cobalt selenide catalysts for the overall water-splitting reaction. For instance, Masud et al. reported Co_7_Se_8_ nanostructured materials as a highly active and stable bifunctional electrocatalyst for both OER and HER in strong alkaline medium [13]. Numerous other cobalt-based electrocatalysts have also been explored; CoSe nanosheets [14], Co_3_Se_4_ nanowires on cobalt foam [15], CoSe_2_ nanosheets [16], and some nonstoichiometric cobalt selenides such as Co_0.85_Se [17] have been reported as overall water-splitting electrocatalysts in alkaline media. Furthermore, the Co_0.85_Se nanosheet network arrayed on a cobalt plate substrate was also reported for HER in both basic and acidic media exhibiting an outstanding catalytic performance, due to its inherent metallic character, giving it abundant surface active sites as well as higher conductivity [18,19]. 

In this family of electrocatalysts, it has been observed that the catalytic activity increases with decreasing electronegativity of the lattice anion. This has been confirmed in many transition metal chalcogenides as OER catalysts whereby changing the anion from O down to Te in the chalcogen group led to significant improvement of the intrinsic catalytic properties in nickel-based chalcogenides [20,21]. Changing the anion leads to decrease in electronegativity in the following order: O (3.44) > S (2.58) > Se (2.55) > Te (2.1), which subsequently leads to increase in the covalency of the metal–chalcogen (M–E) bonds down the chalcogens group, i.e., M–O bonds being less covalent than M–Se or M–Te. This increased covalency assists in local electrochemical oxidation–reduction of the transition metal center by reducing their redox potential as well as by altering the electron density in the catalytically active transition metal site. Increased covalency in the chalcogenides also leads to alteration of the electronic band structure and proper alignment of the valence and conduction band edges with the water oxidation/reduction levels, respectively, leading to more facile charge transfer at the electrocatalyst–electrolyte interface which subsequently reduces the overpotential required for the electrochemical conversion [22,23]. Similar effects have also been observed by doping in transition metal sites which also redistributes electron density around the catalytically active site [24]. 

Until now, the most common cobalt–seleno-based electrocatalysts reported are from different types of nanostructured materials and solids with infinite Co–Se bonds throughout the lattice. Although some of them have shown both efficient catalytic activity for OER and great stability [13,15], there are still some concerns about their stability in alkaline media. For instance, it has been suspected that in alkaline medium these transition metal chalcogenides hydrolyze to form surface oxide layers which act as the actual electrocatalyst in such cases. However, another opposing view is that the transition metal chalcogenides are only partially hydrolyzed, resulting in mixed anionic surface compositions. Several research groups are trying to accurately identify the active surface composition of these transition metal chalcogenide-based electrocatalysts. In that respect, studies of molecular complexes in which the core of the complex represents a structural motif of the metal–chalcogenide solid can shed more light on the effect of anion coordination on the catalytic activity as well as stability of this motif under alkaline conditions. The electronic structure of these molecular complexes can be finely tuned by modifying the electronic and steric properties of the ligands employed in the synthesis. Moreover, the molecular complexes can be very stable, diverse and can exhibit coordination expansion due to ligation of the solvent or other molecules [25,26,27,28,29], which is very useful for the catalytic pathway typically initiated by hydroxide coordination to the catalytically active transition metal site. These properties are of especially great interest for electrochemical H_2_ and O_2_ generation. Therefore, investigating the intrinsic catalytic activity of transition metal complexes with limited or no propensity to form surface metal oxides, as well as understanding the inherent activity of the core structural motif is of paramount interest. Numerous molecular cobalt complexes based on different types of ligand designs have been reported as electrocatalysts [30,31,32,33,34,35,36,37,38,39]. For instance, pentadentate N-heterocyclic coordinated cobalt complexes exhibit HER activity in water [30]. In addition, a cobalt–polypyridyl complex bearing pendant bases and redox-active ligands, combining stability and appropriate redox potential, has also been reported for good electrocatalytic activity in HERs [36]. 

Cobalt-based coordination complexes have previously been studied for OER catalytic activity [37], in instances where the catalysts are immobilized onto the electrode as composite or onto the surface [38]. However, cobalt coordination complexes with seleno-based ligands have not yet been investigated for OER catalytic activity. Such complexes containing the **CoSe_4_** core that is commonly found in cobalt selenide phases will be very useful in understanding the electrocatalytic activity as a function of anion coordination (composition and geometry). This will also aid in gaining an insight into the electrocatalysis pathways and the reasons for significantly enhanced activity of the metal chalcogenides for OER and HER. Herein, we report the bifunctional electrocatalytic activity of a seleno-based Co(II) bis(diselenoimidodiphosphinato) complex, [Co{(SeP^i^Pr_2_)_2_N}_2_], which shows significantly enhanced efficiency for OER and moderate HER, in alkaline medium. A very low onset potential of 1.44 V for O_2_ evolution as well as an overpotential of 320 mV at 10 mA cm^−2^ were recorded. The onset potential for H_2_ evolution is comparable to that of other non-platinum based HER electrocatalysts. 

## 2. Results

**Figure 1:**
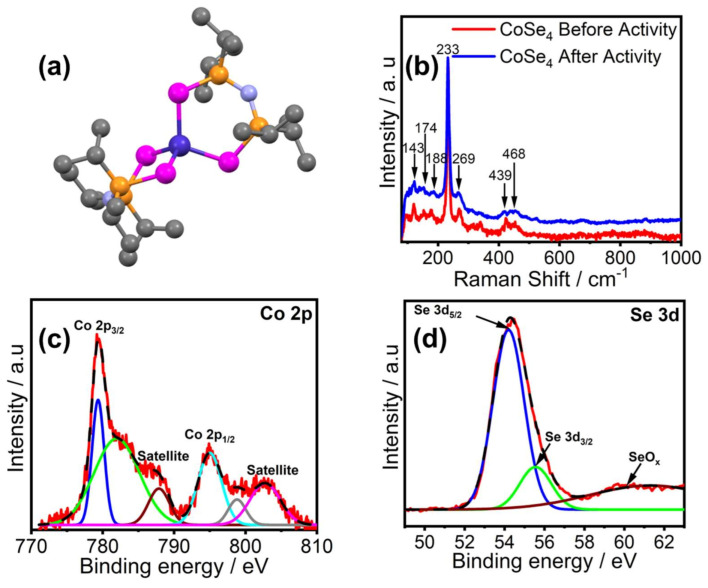
(**a**) The molecular structure of the [Co{(SeP^i^Pr_2_)_2_N}_2_] complex (**CoSe_4_** catalyst), showing the tetrahedral cobalt–seleno-coordination. Color coding: Co (dark blue), Se (magenta), P (brown), N (light blue), C (gray). (**b**) Raman spectra of the complex measured before and after OER catalytic activity. (**c**) Co 2p XPS peaks. (**d**) Se 3d XPS peaks.

The structural and magnetic properties of [Co{(SeP^i^Pr_2_)_2_N}_2_] (referred to as **CoSe_4_** hereafter) have already been reported [39,40]. The crystal structure of **CoSe_4_** shows a first coordination sphere consisting of a Co(II) center tetrahedrally coordinated to four Se atoms stemming from two [SeP^i^Pr_2_)_2_N]^−^ chelating ligands, as shown in Figure 1a. This complex is structurally similar to the Ni(II) tetrahedral NiSe_4_-containing analogue [41,42,43,44]. The complex was further investigated by Raman spectroscopy (Figure 1b) whereby the characteristic peaks observed at 174 and 188 cm^−1^ could be associated to CoSe_2_-like vibrations, while that at 143 and 233 cm^−1^ could be attributed to the trigonal-Se^0^ mode; furthermore, the two peaks at around 444 and 468 cm^−1^ could be associated with the cubic CoSe_2_-like phase mode [45,46], indicating the presence of Co–Se in the **CoSe_4_** complex. The **CoSe_4_** complex was also characterized through X-ray photoelectron spectroscopy (XPS) which showed Co 2p^3/2^ and 2p^1/2^ peaks at 781.2 and 796.3 eV, respectively, characteristic of Co(II), while the Se 3d peaks were observed at 55.1 eV corresponding to Se^2−^ (Figure 1b,c) [47]. The satellites peaks each found at the higher energy end of the Co 2p signals, are attributed to the contribution from antibonding orbital [48]. 

**Figure 2:**
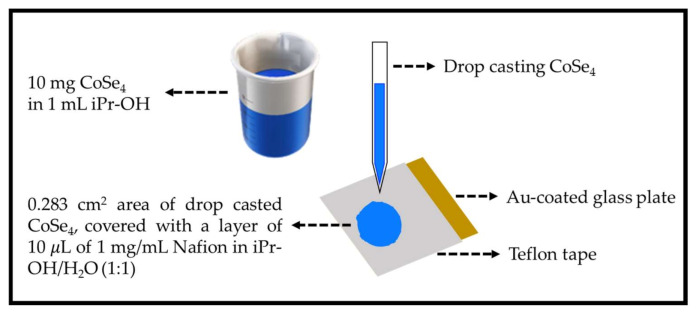
Scheme for electrode preparation.

In order to evaluate the electocatalytic actvity of **CoSe_4_** for water splitting, the drop-casting approach was used to prepare the working electrodes. Specifically, the as-prepared **CoSe_4_** powder was dispersed in isopropyl alcohol (IPA) under ultrasonication without changing its intrinsic properties, and the film was fabricated by gradually dropping the catalyst ink (20 µL) onto Au-coated glass as the conductive substrate over a well-defined geometric area. An amount of 0.1% Nafion was dropped on the top of the coating to form a covering layer as illustrated in Figure 2. This method has been widely used in our previous studies for preparing electrode material from powder catalyst samples [44]. Electrodes were also prepared with glassy carbon (GC) substrates. The electrocatalytic activity of **CoSe_4_** was compared with RuO_2_ electrodes prepared on Au-coated glass and GC following the above procedure.

**Figure 3:**
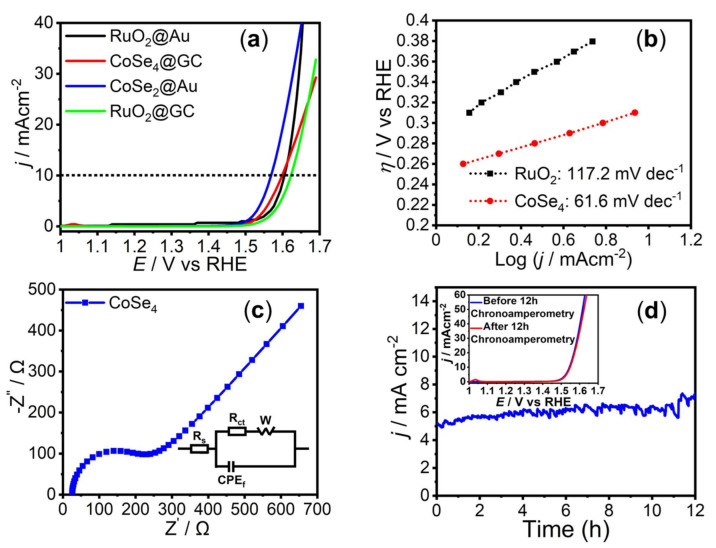
(**a**) LSV plots of OER for the **CoSe_4_** catalyst measured in N_2_-saturated 1.0 M KOH solution at a scan rate of 10 mV s^−1^. Dashed black line shows the current density of 10 mA cm^−2^. (**b**) Tafel plots of catalysts. (**c**) EIS spectra measured at 1.56 V vs. RHE in N2-saturated 1.0 M KOH solution over the frequency range of 1 MHz to 1 Hz. Inset shows the equivalent circuit where R_s_ is the electrolyte resistance and R_ct_ is the charge transfer resistance at the catalyst–electrolyte interface. (**d**) Stability study of **CoSe_4_** catalyst under continuous O_2_ evolution for 12 h at 1.53 V. Inset shows the LSV plots of the **CoSe_4_** catalyst in N_2_ saturated 1.0 M KOH before (blue) and after (red) chronoamperometry for 12 h.

The OER catalytic process was studied by linear sweep voltammetry (LSV) measurements conducted in N_2_-saturated 1 M KOH, at a scan rate of 10 mV s^−1^. Figure 3a shows the LSV plots of electrochemical oxygen evolution at **CoSe_4_**@Au, **CoSe_4_**@GC (GC: glassy carbon), RuO_2_@Au and RuO_2_@GC electrodes. **CoSe_4_** loaded on Au-glass and GC electrodes showed high activity for OER with the exchange current density corresponding to O_2_ evolution showing a sharp and slow increase for Au and GC, respectively. Among these **CoSe_4_**@Au-glass showed lower onset potential for OER implying that using the Au-coated glass as primary electrode for the **CoSe_4_** catalyst, the latter shows higher activity toward OER compared to a GC electrode. The typical performance parameters including onset and overpotentials for the **CoSe_4_**@Au-glass catalyst evaluated in this work are listed in Table 1 below.

**Figure 4:**
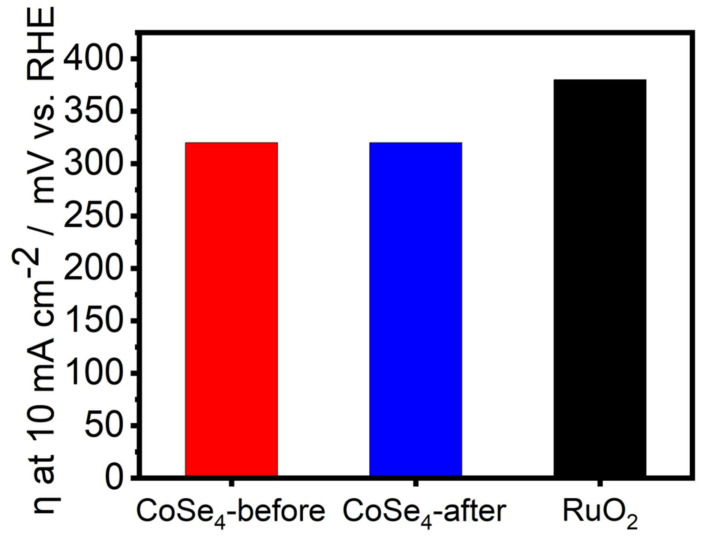
Comparison of achieved overpotentials at 10 mA cm^−2^ for **CoSe_4_** compared with RuO_2_.

The onset potentials for **CoSe_4_**@Au-glass and RuO_2_@Au-glass were 1.44 and 1.51 vs. reversible hydrogen electrode (RHE), respectively, as shown in Figure 3a. The overpotential required to achieve current density of 10 mA cm^−2^ (considering the electrode geometric area) for **CoSe_4_**@Au-glass and RuO_2_@Au-glass were 320 and 380 mV, respectively (Figure 3a and Figure 4). This indicates the superiority in catalytic activity of **CoSe_4_** compared to state-of-the-art (RuO_2_) OER electrocatalyst (Figure 4). Furthermore, Tafel slopes obtained from Tafel plots (*η* vs. log *j*) applying Equation (2), were used to investigate OER kinetics of the **CoSe_4_**@Au-glass and RuO_2_@Au-glass electrodes, as shown in Figure 3b. Tafel slopes of 61.6 and 117.2 mV dec^−1^ were obtained for **CoSe_4_**@Au-glass and RuO_2_@Au-glass, respectively. The smaller Tafel slope shown by **CoSe_4_**-modified electrodes compared to RuO_2_ further confirms faster kinetics for OER and consequently better electrocatalytic efficiency of **CoSe_4_**. The Tafel slope for **CoSe_4_** is also comparable to reported values for other transition-metal–chalcogenide-based catalysts [14,24]. The **CoSe_4_**@Au-glass was also analyzed through electroimpedance spectroscopy (EIS) to estimate the charge transfer resistance (R_CT_) at the catalyst–electrolyte interface. The EIS spectra shown in Figure 3c could be fitted to an equivalent circuit and the R_CT_ was estimated to be approximately 180 ohm. The low value of R_CT_ indicates faster charge-transfer at the catalyst interface. 

**Figure 5:**
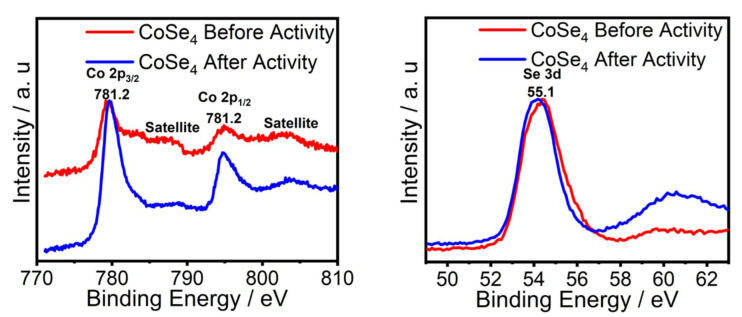
Comparison of XPS peaks of Co 2p and Se 3d before and after OER activity confirming the stability of the **CoSe_4_** catalyst.

In addition, the mass activity and turn-over frequency (TOF) of the **CoSe_4_**@Au catalyst was calculated at an *η* value of 0.320 V (Table 1). The mass activity was estimated to be 14.15 A g^−1^, indicating better performance than some of the state of the art OER electrocatalysts, such as IrO_x_ and RuO_x_ [49]. The OER TOF at an overpotential of 320 mV for the **CoSe_4_**@Au-glass catalyst was estimated to be 0.032 s^−1^, under the assumption that all metal ions in the catalysts are catalytically active (Equation (3)). However, since not each and every metal atom is expected to be involved in the reaction, the actual TOF could be evidently underestimated. However, the calculated TOF for **CoSe_4_**@Au-glass is still important (Table 1), and is comparable or higher than that of other cobalt-based catalysts previously reported under similar conditions [50,51]. The stability of the **CoSe_4_**@Au-glass electrode under long-term electrolysis in alkaline medium was also investigated using the chronoamperometric technique by which the current density was measured at a constant applied potential of 1.53 V vs. RHE for prolonged period of time, as shown in Figure 3c. The stable current density over 12 h of continuous oxygen evolution indicates high functional durability exhibited by the **CoSe_4_**@Au-glass catalyst for OER in 1 M KOH. The LSV plots after 12 h of chronoamperometry (inset of Figure 3c) show no noticeable difference with the pristine catalyst, except for the small peak at 1.03 V (RHE) indicating the partial oxidation of Co(II) to Co(III) [52]. Interestingly, the LSV plots confirmed that there was no degradation of catalyst performance for OER under conditions of continuous O_2_ evolution for an extended period of time. The stability of the **CoSe_4_** catalyst was also confirmed with XPS measured after 12 h chronoamperometry measurement as shown in Figure 5. The XPS spectra showed the presence of Co 2p and Se 3d peaks with similar peak positions. The comparison of XPS spectra before and after OER activity showed no noticeable change as shown in Figure 5 confirming that the **CoSe_4_** complex was indeed stable.

**Figure 6:**
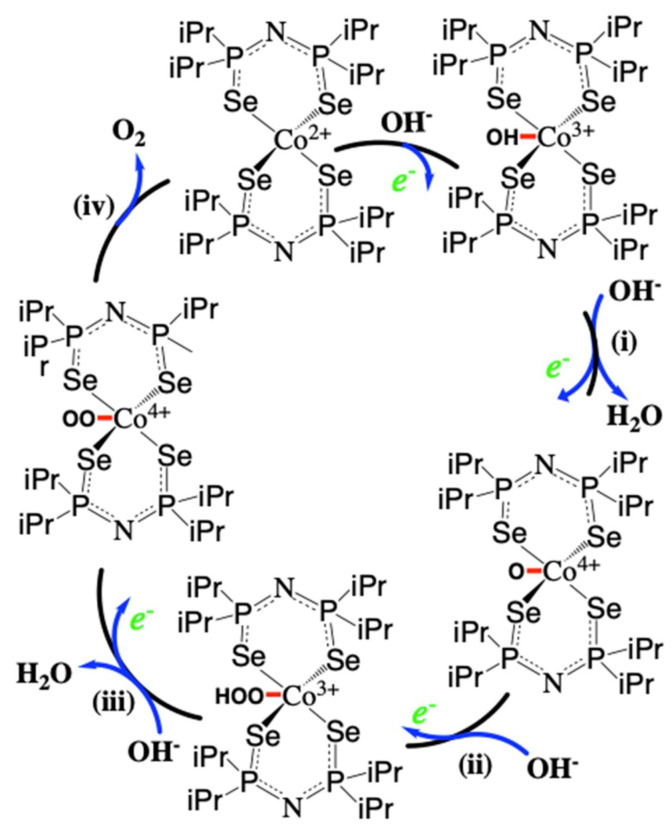
Schematic illustration of the proposed OER mechanism on the **CoSe_4_** complex through a vacant metal coordination site for OER showing the formation of a five-coordinated square pyramidal transition state following the catalyst’s activation through -OH^−^ coordination to Co(II) and subsequent steps of OER.

This efficient OER electrocatalytic activity observed for the **CoSe_4_** catalyst is a significant step in an effort to understand the inherent OER catalytic performance of transition metal selenides. It is understood that the stability of the M*E*_4_ center (M = metal, *E* = chalcogen) increases with decreasing electronegativity of the chalcogen atom, *E* [22]. The OER catalytic process is expected to be initiated by coordination of the OH^−^ group to the catalytically active Co(II) center. Tetrahedral Co(II) complexes containing chalcogenated imidodiphosphinato ligands have the tendency to increase their coordination number from four to six in the presence of coordinated solvents such as DMF, [29]. Consequently, the catalytic process may be initiated by OH^−^ coordination to the metal center without cleavage of the Co–Se bonds through coordination expansion, where the Co(II) center is concomitantly oxidized to Co(III) in order to accommodate the extra anionic charge. This proposition is also supported by the Tafel slope, which shows a value less than 120 for the **CoSe_4_** catalyst (Table 1). The lower Tafel slope suggests that the rate-determining step in the catalytic process corresponds to subsequent electron transfer steps from the catalyst’s surface to the electrolyte, rather than the initial OH^−^ coordination to Co(II). Even though there are very scant reports on the proposed mechanisms specific to tetrahedral cobalt-based complexes [53,54], a mechanism proposed for similar transition metal-based complexes has been reported [55]. Hence, it can be postulated that the tetrahedral **CoSe_4_**-containing complex is further coordinated to one OH^−^/H_2_O group leading to the formation of a square pyramidal transition state, which can then react further to form an O–O linkage, and a subsequent removal of O_2_ (Figure 6). The Co site undergoes a reversible change in oxidation states from +2 to +3 and +4 following adsorption of the anionic intermediates and formation of the transition states. The observation of Co oxidation peak in the LSV (Figure 3c inset) supports the formation of higher oxidation states of Co during the catalytic cycles providing some support to this proposed mechanism. However, it must be mentioned here that this proposed mechanism is based on the general scheme of multi-step proton coupled electron transfer mechanism for OER that has been observed and reported for other electrocatalytic systems [53,54]. To decipher the actual mechanism, one needs to identify the transition states through in situ spectroscopy and other techniques. However, based on characterization of the catalyst after OER activity, it can be clearly seen that the **CoSe_4_** catalytic core remains intact and maintains its Co-Se bonds and, therefore, even though the catalytic reaction is initiated by coordination of OH^−^ to the Co(II) center, the complex is not converted into any form of oxide/hydroxide. This confirms that the **CoSe_4_** core is indeed stable for OER and hence the mechanism proceeds via formation of a mixed anionic (hydroxo)chalcogenide coordination. This mechanistic scheme could be extrapolated to the cobalt–selenide-based extended solids that have been reported as active electrocatalysts for OER. 

**Figure 7:**
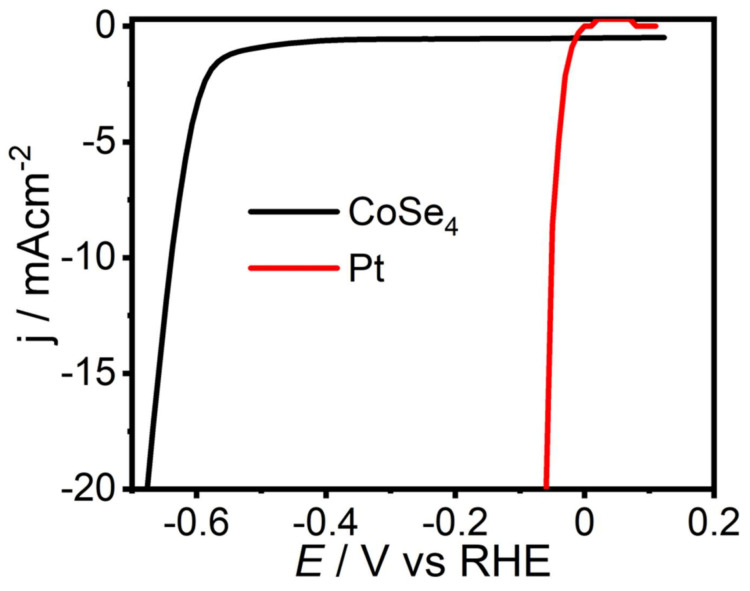
Polarization curves for HER with the **CoSe_4_** catalyst in N_2_-saturated 1.0 M KOH solution at a scan rate of 10 mV s^−1^.

The electrocatalytic HER performance of the **CoSe_4_** catalyst and that of the state-of-the-art Pt catalyst were also studied and compared under similar conditions (in N_2_-saturated 1 M KOH). In order to avoid any form of catalytic activity interference, the HER activity of **CoSe_4_** was evaluated using GC as the counter electrode instead of Pt, since Pt could undergo anodic dissolution and redeposit onto the cathode, which would affect the activity. The LSV (Figure 7) obtained with **CoSe_4_**@Au-glass as cathode confirmed that it is active for HER and showed an onset potential of 0.52 V, which was higher than that with a Pt cathode. The cathodic current increased rapidly under more negative potentials. The overpotential for **CoSe_4_**@Au at a current density of 10 mA cm^−2^ was 0.63 V, which is also far from that with a Pt cathode. This shows that **CoSe_4_** is moderately active for HER. The catalytic activity can be possibly improved by intermixing the electrocatalyst with other conducting additives such as activated carbon, graphene or carbon nanotubes as has been reported earlier for other catalysts [56,57,58].

## 3. Materials and Methods

### 3.1. Synthesis of bis(diselenoimidodiphosphinato) Cobalt(II) Complex [Co{(SeP^i^Pr_2_)_2_N}_2_]

The synthesis of the (SeP^i^Pr_2_)_2_NH ligand [59] and the [Co{(SeP^i^Pr_2_)_2_N}_2_] complex [39] was carried out following previously reported procedures.

### 3.2. Electrochemical Measurements 

In order to study the OER and HER catalytic activity, the **CoSe_4_** catalyst was mixed with nafion and drop-cast onto different electrodes. All the electrochemical measurements were investigated using an electrochemical workstation (IvumStat potentiostat) in a standard three-electrode cell, with N_2_-saturated 1 M KOH as the electrolyte solution. For all measurements, Ag/AgCl was used as reference electrode, while GC and Pt mesh served as counter electrodes for HER and OER, respectively. Catalyst loaded on Au or GC served as the working electrode. The LSVs were performed at a scanning rate of 10 mV s^−1^ while the electrode was rotating at 1000 rpm. In order to reduce uncompensated solution resistance, all activity data were iR corrected, which was measured through electrochemical impedance studies. 

The reference electrode was calibrated by measuring open circuit potential (OCP, −0.199 V) at Pt wire in pure H_2_-saturated 1.0 M H_2_SO_4_ solution. Potentials measured vs. Ag/AgCl electrode were converted to values vs. RHE on the basis of Nernst’s equation (Equation (1)):

𝐸_RHE_ = 𝐸_Ag/AgCl_ + 0.059pH + 𝐸^0^_Ag/AgCl_ (1)

where *E*_RHE_ is the converted potential vs. RHE, *E*_Ag/AgCl_ is the experimentally measured potential against the Ag/AgCl reference electrode, and 𝐸^0^_Ag/AgCl_ is the standard potential of Ag/AgCl at 25 °C (0.199 V). 

### 3.3. Tafel Plots

The Tafel slope was calculated by applying Equation (2):

*η* = a + (2.3RT/𝛼nF) log(*j*) (2)

where *η* is the overpotential, *j* is the current density, and the other symbols have their usual meanings. The Tafel equation as shown Equation (2) is a fundamental equation which is acquired from the kinetically control region of OER/HER and relates the overpotential *η* with the current density *j* where the Tafel slope is given by 2.3RT/αnF. 

### 3.4. Turnover Frequency (TOF)

The TOF value was calculated by Equation (3):

TOF = I/(4 × F × M)(3)

where I is the current in Ampere, F is the Faraday constant and M is the number of moles of the active catalyst. 

### 3.5. Electrode Preparation

Au-coated glass and GC used as substrates were purchased from Deposition Research Lab Incorporated (DRLI), Lebanon Missouri and Fuel Cells Etc, Texas, respectively. All solutions were prepared using deionized (DI) water. All substrates were cleaned by isopropanol and eventually rinsed with deionized water to ensure a clean surface. Catalyst ink was prepared by dispersing 10.0 mg of the catalyst in 1.0 mL isopropyl alcohol (IPA) and ultrasonicated for 30 min. Au-coated glass substrate were covered with a Teflon tape, leaving an exposed geometric area of 0.283 cm^2^ and GC (geometric area: 0.196 cm^2^) served as the working electrodes. A quantity of 20 μL of the ink was pipetted out on the top of the Au or GC substrates. The catalyst layer was dried at room temperature. Then, an aliquot of Nafion solution (10 μL of 1 mg/mL solution in 50% IPA in water) was applied onto the catalyst layer. The Nafion-coated working electrode was dried at room temperature and finally heated at 130 °C in an oven for 30 min in air.

### 3.6. Electrodeposition of RuO_2_

Electrodeposition of RuO_2_ on Au-coated glass and GC substrate were carried out from a mixture of RuCl_3_ (0.452 g) and KCl (2.952 g) in 40 mL of 0.01 M HCl using cyclic voltammetry from 0.015 to 0.915 V (vs. Ag|AgCl) for 100 cycles at a scan rate of 50 mV s^−1^. This was then heated in ambient air for 3 h at 200 °C. 

## 4. Conclusions

A cobalt–seleno-complex, [Co{(SeP^i^Pr_2_)_2_N}_2_], was reported as a bifunctional catalyst for OER and HER in alkaline medium. This complex bearing a catalytically active **CoSe_4_** first coordination sphere shows high inherent catalytic activity for OER as evidenced by the low overpotential and Tafel slope. This observed activity can be attributed to the higher covalency of the metal–chalcogen bond in **CoSe_4_** relative to cobalt oxides, which explains the observed enhancement in catalytic efficiency. The **CoSe_4_** complex demonstrated good OER activity in 1.0 M KOH with an overpotential of 320 mV at 10 mA cm^−2^. Even though the activity for HER in alkaline medium demonstrated by the complex was low, modifying the ligands with either electron density donating or withdrawing groups [59,60] may improve HER activity. The superior catalytic activity shown by **CoSe_4_** for OER, as well as its remarkable stability, indicates its promising potential as a noble-metal-free catalyst for OER in water splitting. Further work on the ligand modification and elucidation of the mechanism and kinetics involved, as well as photoelectrocatalytic water splitting using this type of MSe_4_-containing catalysts in the presence of photosensitizers is under investigation. 

## Figures and Tables

**Table 1:** Electrochemical parameters of the catalysts measured in 1 M NaOH.

OER						HER	
Catalyst	Onset Potential (V) ^1^	η to 10 mA cm^−2^ (V) ^1^	Tafel Slope (mV dec^−1^)	Mass Activity at 320 mV (A g^−1^)	TOF at 320 mV (s^−1^)	Onset Potential (V) ^1^	η to 10 mA cm^−2^ (V) ^1^
**CoSe_4_**@Au	1.44	0.32	61.6	14.15	0.032	0.52	0.63
RuO_2_@Au ^2^	1.51	0.38	117.2	-	-	-	-
Pt	-	-	-	-	-	0.00	0.05

^1^ Potential vs. RHE, ^2^ electrodeposited.

## Data Availability

The data presented in this study are available on request from the corresponding author. The data are not publicly available due to this being part of federally funded research.

## Sample Availability

Samples of the compounds are available from the authors on request.
